# Improving the field accuracy of a malaria diagnostic algorithm combining sequential interpretation of rapid diagnostic test detecting *Pf*HRP2 and *p*LDH in febrile children in a seasonal hyperendemic malaria transmission area in Burkina Faso

**DOI:** 10.1371/journal.pone.0351990

**Published:** 2026-06-23

**Authors:** Diane Yirgnur Some, Francois Kiemde, Berenger Kabore, Daniel Valia, Toussaint Rouamba, Seydou Sawadogo, Athanase M. Some, Hermann Sorgho, Macaire Nana, Yacouba Nombre, Nadine A. Kone, Adelaide Compaore, Fadima Yaya Bocoum, Massa dit Achille Bonko, Georges Some, Gautier Tougri, Sylvie Yeri Youl, Konseibo Noellie, Yeri Esther Hien, Aly Savadogo, Fla Koueta, Henk D. F. H. Schallig, Halidou Tinto

**Affiliations:** 1 Institut de Recherche en Sciences de la Santé - Clinical Research Unit Of Nanoro (IRSS-CRUN), Ouagadougou, Burkina Faso; 2 Université Joseph Ki-Zerbo Ouaga 1, Unité de Recherche et de Formation en Sciences de la Vie et de la Terre (UFR-SVT), Ouagadougou, Burkina Faso; 3 Health District of Nanoro, Ministry of Health, Nanoro, Burkina Faso; 4 National Malaria Control Program, Ministry of Health, Ouagadougou, Burkina Faso; 5 National Agency for Primary Healthcare, Ministry of Health, Ouagadougou, Burkina Faso; 6 Department of Pediatrics, CHU Yalgado Ouedraogo, Ouagadougou, Burkina Faso; 7 Department of Medical Microbiology and Infection Prevention, Laboratory for Experimental Parasitology, Amsterdam University Medical Centre, Amsterdam, The Netherlands; 8 Amsterdam institute for Immunology and Infectious Diseases, Amsterdam, The Netherlands; 9 Amsterdam Institute for Global Health and Development, Amsterdam, The Netherlands; University of Health and Allied Sciences, GHANA

## Abstract

**Objective:**

**To e**valuate the field accuracy of a malaria diagnostic algorithm combining sequential interpretation of two-step malaria RDT detecting *Pf*HRP2 and *p*LDH with information on previous antimalarial treatments within the past four weeks for the diagnosis of malaria in febrile children under 5 years compared to standard diagnosis using a *Pf*HRP2 only based RDT.

**Methods:**

Febrile children aged 6–59 months attending outpatient clinics were randomized to either the control group, which received the standard RDT (*Pf*HRP2 only), or the intervention groups (an e-algorithm or a decisional algorithm), which was subjected to the diagnostic algorithm combining an RDT detecting *Pf*HRP2 and *p*LDH with information on previous antimalarial treatment. Malaria diagnosis with *Pf*HRP2-based RDT was reported as positive or negative. The sequential interpretation was reported as (i) positive when the *p*LDH line appeared, regardless of the *Pf*HRP2 results, (ii) negative when both lines did not appear and (iii) undetermined when only the *Pf*HRP2 line appeared, and information on previous antimalarial treatment within the past 4 weeks was used as a decision-support tool to classify active malaria from past infection. Blood samples were also collected for expert microscopy as the gold standard, and for qPCR to further evaluate undeterminate results and potential false-positive RDT outcomes.

**Results:**

In total 1176 children were included, with 66.7% (784/1176) assigned to the intervention arms and 33.3% (392/1176) to the control arm. In patients assigned to the sequential algorithm, the number of undetermined cases was 12.7% (100/784). Considering microscopy as the gold standard, *Pf*HRP2-based RDT reported a sensitivity of 96.5% and a of specificity 79.1%, with positive and negative predictive values of 78.3% and 96.7%, respectively. For the sequential algorithm, the sensitivity, specificity, positive and negative predictive values of the conclusive-only results (i.e., *Pf*HRP2±/*p*LDH+ and *Pf*HRP2-/*p*LDH-) were 97.4%, 98.4%, 98.0% and 97.9%. However, when undetermined result were combined with conclusive results, the sensitivity, specificity, positive and negative predictive values were 89.7%, 96.8%, 95.6% and 92.4% respectively. Among recently antimalarial treated participants in sequential algorithm arm, 59.5% (50/84) were qPCR-positive, compared to 68.7% (11/16) qPCR-positivity in those without recent treatment.

**Conclusions:**

The sequential diagnostic approach improves the diagnosis of malaria in a real world setting, compared to the use of *Pf*HRP2-(only) based RDT. However, relying only on history of antimalarial treatment in undetermined cases may decrease algorithm’s sensitivity, which could result in missing active or recurrent malaria infections.

## Introduction

Malaria rapid diagnostic tests (RDT) are essential tools in the management of febrile diseases in resource-limited settings without routine microscopy [1]. Malaria RDTs are simple, affordable, rapid and easy to perform by non-technicians and do not require sophisticated laboratory infrastructure, making them widely available to front-line healthcare workers at the point-of-care and allowing for timely treatment decisions [2]. However, the effective use of malaria RDTs in routine health systems is hindered due to their limitations, such as the persistence of *Plasmodium falciparum* histidine rich protein-2 (*Pf*HRR2) antigen up to four weeks after successful treatment and the low sensitivity of *Plasmodium* Lactate Dehydrogenase (*p*LDH) -based tests [3]. These limitations can result in misdiagnosis of the real cause of fever [4].

To overcome this issue, a diagnostic algorithm combining sequential interpretation of *Pf*HRP2/*p*LDH RDT with information on previous antimalarial treatment within the past two weeks has previously been proposed [5]. The findings of this study demonstrated that this sequential approach significantly reduced the rate of incorrect diagnoses among febrile children compared to the single *Pf*HRP2-based RDT. The positive predictive value was improved (90% for the sequential interpretation *versus* 84% for *Pf*HRP2 antigen), highlighting the diagnostic benefit of the sequential diagnostic approach in the context of high malaria transmission.. While the advantages of combining *Pf*HRP2 and *p*LDH in sequential interpretations to diagnose malaria were obvious, the challenge remained to differentiate malaria cases in patients with undetermined results; i.e., cases with *Pf*HRP2 + /*p*LDH- results. These results are either due to persisting *Pf*HRP2 antigen after successful antimalarial treatment, or the low sensitivity of the *p*LDH-based RDT [5]. In the previous study, information on prior antimalarial treatment within the past two weeks was used as a decision-support tool to classify active malaria infection from past infection in patients with undetermined (*Pf*HRP2 + /*p*LDH-) results [1,4,6]. It allowed for thedetection of a portion of false-positive results (mainly) due to the *Pf*HRP2 antigen persistence. In light of these findings, we hypothesize that including information on prior antimalarial treatment within the past four weeks will further increase the diagnostic accuracy [4,7–9].

This diagnostic approach was evaluated within a three-arm randomized controlled trial designed to improve the management of febrile illnesses and address antimicrobial resistance. Two intervention arms comprising: 1) the e-algorithm which is managing participants using the enhanced diagnostic algorithm integrating *Pf*HRP2/*p*LDH results with information on previous antimalarial treatment (diagnostic and treatment are guided by designed clinical electronic descision support platform), and 2) the decisional algoritm arm in which participants are managed according to the predefined diagnostic decision framework without electronic integration (diagnostic and treatment are left to the discretion of the healthcare workers). The control arm comprised routine care practices and uses a single-step *Pf*HRP2 RDT as diagnostic test [10]. The purpose of the present study was to assess the accuracy of a malaria diagnosis approach tested combining sequential interpretation of two-step RDTs detecting *Pf*HRP2 and *p*LDH in children under 5 years compared to routine RDT detecting *Pf*HRP2.

## Methods

### Study design

**Fig 1:**
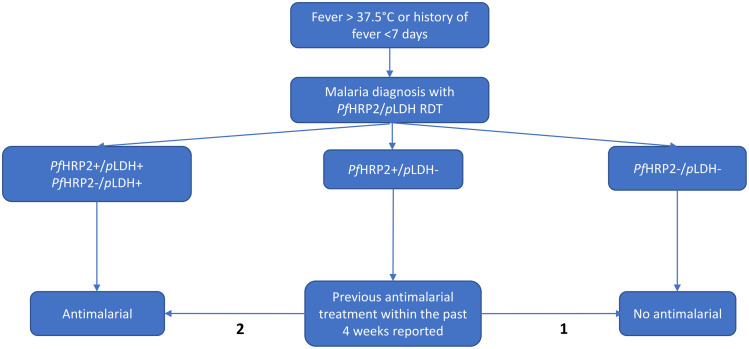
Proposed sequential algorithm workflow combining the two-step malaria RDT detecting *Pf*HRP2/*p*LDH and information on previous antimalarial treatment within the past 4 weeks: Judgement of malaria infection in undetermined cases: 1: Yes = no antimalarial treatment (no malaria)m 2: No=antimalaria treatment (malaria).

A prospective, comparative, randomized controlled trial with 3-arms (2 intervention arms, which include i) an e-algorithm or ii)a decisional algorithm, and 1 control arm [standard practise]), was conducted from 4 March 2022–28 February 2023 at the outpatient clinic of Bologho in the health district of Nanoro (Burkina Faso). The aim of the study was to evaluate the algorithm combining two-step RDT detecting *Pf*HRP2/*p*LDH compared to single *Pf*HRP2-based RDT for the diagnosis of malaria in febrile children [10]. Briefly, all children aged 6–59 months attending the health facility with axillary temperature ≥37.5°C or history of fever within the past 7 days were eligible. Written informed consent was obtained from the parents/guardians prior to any data or clinical sample collection. Participants were randomised either to the control arm, which followed the routine care pathway including *Pf*HRP2-based RDT or to one of the two intervention arms, in which the participants were subjected to an enhanced diagnostic package including the sequential interpretation combining the two-step RDT detecting *Pf*HRP2/*p*LDH and information on previous antimalarial treatment within the past 4 weeks (i.e., e-Algorithm and decisional algorithm described in Fig 1). Fingerprick capillary blood (around 200 µL) was collected in a microtube EDTA for microscopy and dried blood spot (DBS) preparation on Whatman 3 filter paper for qPCR testing to further assess undetermined results and false-positive RDT results.

Malaria case management in the study area followed the Burkinabe national guidelines aligned with World Health Organization (WHO) recommendations, including parasitological confirmation by microscopy or RDTs prior to treatment, and the use of artemisinin-based combination therapies (ACTs) as first-line treatment for uncomplicated malaria. At the time of the study, available data indicated that the prevalence of *pfhrp*2/*pfhrp*3 gene deletions in the study area was below the 5% threshold recommended by WHO for reconsideration of *Pf*HRP2-based RDT use [11,12].

### Laboratory procedures

#### Malaria rapid diagnostic test.

The *Pf*HRP2-based RDT (SD Bioline Pf: Standard Diagnostic, Hagal-Dong, Republic of Korea and the two-step (double band) malaria RDT (SD Bioline® Malaria Ag Pf/Pan batches 05EDG063A, 05EDG030A: Standard Diagnostics, Hagal-Dong, Republic of Korea), were performed at primary health centers by trained nurses. For both tests, five (5) μl of capillary blood sample was used.

The results of the *Pf*HRP2-based RDT were recorded as positive or negative.

To evaluate the accuracy of the proposed algorithm combining *Pf*HRP2 and *p*LDH, with information on previous antimalarial treatment within the past four weeks, the diagnostic results of the *Pf*HRP2 and *p*LDH antigens were recorded followed by previous antimalarial treatment results when undetermined. The interpretation of the two-step RDT detecting *Pf*HRP2 and *p*LDH was based on local epidemiology and done as follows (Fig 1):

- *Pf*HRP2(+)/*p*LDH(+): when both lines appeared in the test, the malaria diagnosis is considered positive to *falciparum* malaria or a possible co-infection with non-*falciparum* malaria;
- *Pf*HRP2(-)/*p*LDH(+): when only *p*LDH line appeared, the malaria diagnosis is considered as positive and more likely indicate a non-*falciparum* malaria infection or *falciparum* malaria with a potential *hrp2* gene deletion;
- *Pf*HRP2(-)/*p*LDH(-): when no line appeared, malaria results is negative
- *Pf*HRP2(+)/*p*LDH(-):when only the *Pf*HRP2 line appeared, malaria test result is undetermined and information on previous antimalarial treatment was used as a decision-support tool to classify malaria infection:
  - If previous antimalarial treatment is reported within the past 4 weeks (< 28 days), the malaria diagnosis was reported as negative. Nonetheless, for ethical considerations, the antimalarial treatment decision was based on malaria microscopy.
  - If previous antimalarial treatment is not reported within the past 4 weeks (< 28 days), the malaria diagnosis was reported as positive and antimalarial was prescribed.

The workflow of the proposed sequential algorithm is summarized in Fig 1.

All malaria RDT results (i.e., single *Pf*HRP2-based RDT and the sequential algorithm) were independently read by 2 trained nurses. A third reader was requested in case of disagreement.

#### Microscopy.

Malaria diagnosis by microscopy (double reading) was done by expert microscopists at Clinical Research Unit of Nanoro (CRUN) who are subjected to regular external quality control (National Institute of Communicable Diseases/World Health Organization; NICD/WHO). All expert microscopists are certified WHO Level 1, ensuring high level of proficiency and adherence to international quality standard. Thick and thin blood smears were performed in duplicate from EDTA blood samples for each participant, air-dried and stained with 10% Giemsa solution for 20 minutes. Malaria parasitemia was expressed as parasites/μl of blood and calculated by counting the number of asexual parasites per 200 leukocytes (if the count was > 10 asexual parasites) and by 500 leukocytes (if the count was < 10 asexual parasites), assuming a leukocyte count of 8000 per μL of blood. In case of a discrepant result, a third reading was performed. Discrepancy involves positive *versus* negative, differences in *Plasmodium* species, or differences in parasite density (i.e., the difference between two-readings exceeding 50% of the lower reading).

#### DNA extraction and VarATS quantitative PCR.

The qPCR test was performed in case of discrepancy in results between the RDTs (sequential diagnosis and routine RDT) and microscopy, and undetermined results to two-steps malaria RDT. Briefly, malaria parasite DNA (deoxyribonucleic acid) was extracted from DBS using QIAamp DNA mini kit® (Qiagen, Germany) and stored at -20^o^C until the qPCR test was done. A volume of five (5) µL of DNA were used as template for qPCR analysis targeting *P. falciparum* var gene acidic terminal sequence (varATS, ≈ 59 copies per genome) as previously described [13]. The qPCR was run on StepOnePlus (Applied Biosystems™). The parasite densities were obtained by interpolating cycle thresholds (Ct) using a standard curve prepared with titrated samples (100,000–0.1 parasites/μL). The limit of detection of the varATS-based qPCR was 0.4 parasite/μL for DNA extracted from filter paper. Samples with Ct value >38.0 were considered as negative. Stored DNA of *P. falciparum* with a known parasitemia was used as positive control. The negative controls included human negative blood spots on filter paper and master mix reagents used as no template control (NTC).

### Data collection and analysis

Data were collected using electronic Case Report Forms (e-CRFs) built on Android Studio and then uploaded to a secured server hosted and managed by CRUN. Data were extracted in Excel format, cleaned and analysed using STATA software version 17 (StataCorp. Stata Statistical Software: Release 17. College Station, TX: StataCorp LLC; 2021). Qualitative and quantitative variables were described respectively by their frequencies with 95% confidence intervals and by their medians with interquartile ranges. Diagnostic accuracy of the sequential algorithm and the *Pf*HRP2-based RDT was evaluated by calculating their sensitivity, specificity, positive predictive value and negative predictive value using microscopy as the gold standard. Parasite densities were expressed by geometric mean. The level of agreement between the sequential algorithm and microscopy was evaluated by the Cohen’s Kappa values. Standard classification suggested by McHugh [14] has been used to interprete kappa values. A significance threshold of α = 5% was used for all analyses.

## Results

### Baseline clinical and socio-demographic characteristics of the study population

From 4 March 2022–28 February 2023, 1176 children aged 6–59 months were enrolled. Of these patients enrolled, 784 (66.7%) were tested with the sequential algorithm (i.e., 394 and 390 in the decisonal algorithm and e-algorithm arms, respectively) and 392 (33.3%) with *Pf*HRP2-based RDT only as recommended by the local standard routine care. Mono-infections with *Plasmodium falciparum* (*P.falciparum)* were found by expert microscopy in 97.7% (514/526) of participants. The participants’ clinical and socio-demographic features are detailed in Table 1.

**Table 1:** Baseline characteristics of the study population in decisionnel, e-algorithm and control arm (standard routine care).

Characteristics		Study arm		
	Total, N (%)	Decisional, n (%)	e-algorithm n (%)	Control n (%)
	**1176**	**394 (33.5)**	**390 (33.2)**	**392 (33.3)**
**Sex**				
**Male**	580 (49.3)	189 (48.0)	202 (51.8)	189 (48.2)
**Female**	596 (50.7)	205 (52.0)	188 (48.2)	203 (51.8)
**Age median (months) (IQR)**	27.0 (15.0-47.0)	27.5 (16.0-41.0)	26.5 (16.0-42.0)	26.0 (15.0-40.5)
**Age group [months]**				
**[6–11]**	192 (16.4)	62 (15.7)	59 (15.1)	71 (18.1)
**[12–23]**	317 (26.8)	102 (25.9)	114 (29.2)	101 (25.8)
**[24–59]**	667 (56.8)	230 (58.4)	217 (55.6)	220 (56.1)
**Season of consultation**				
Low malaria transmission season*	665 (56.5)	221 (56.1)	221 (56.7)	223 (56.9)
**High malaria transmission season** ^**†**^	511 (43.5)	173 (43.9)	169 (43.3)	169 (43.1)
**Temperature (°C)**				
**< 37.5**	533 (45.4)	200 (50.8)	181(46.4)	152(38.8)
**≥ 37.5**	643 (54.6)	194 (49.2)	209 (53.6)	240 (61.2)
**Malaria cases by expert microscopy**	526 (44.7)	183 (46.4)	167 (42.8)	176 (44.9)
**Malaria parasitaemia/µL blood, (geometric mean)**	24086.4 [17327.52 - 33485.68]	24302.8 [17391.1 - 33961.4]	25640.7 [18280.93 - 35963.37]	22315.9 [16310.55 - 30532.29]
**Non-*falciparum* species**	12 (1.0)	05 (41.7)	02 (16.6)	5 (41.7)

* = November to May; † = June to October. Legend: The e-algorithm group included participants managed using the enhanced diagnostic algorithm (clinical decision support platform) integrating *Pf*HRP2/*p*LDH RDT results with treatment-history–based resolution. The decisional algorithm group included participants managed according to the predefined diagnostic decision framework without electronic integration. The routine group included participants managed using the local guideline integrating only *Pf*HRP2 results. The total number of participants included in the analysis is indicated in the table.

### Diagnostic accuracy of the sequential interpretation or routine *Pf*HRP2 compared to microscopy

Table 2 summarizes the results of the sequantial diagnostic approaches and routine *Pf*HRP2 test, compared to expert microscopy (gold standard). The sequential diagnosis identified 87.3% (684/784) of conclusive cases (*Pf*HRP2 + /*p*LDH + , *Pf*HRP2-/*p*LDH + , or *Pf*HRP2-/pLDH-) and 12.7% (100/784) yielded undetermined (*Pf*HRP2 + /*p*LDH-) results were classified as “undetermined”. The results of *Pf*HRP2-/*p*LDH+ were interpreted as potential non-*falciparum* malaria or *P. falciparum* infection with possible *hrp*2 deletion, consistent with the algorithm characteristics of the two-step malaria RDT used. However, microscopy identified non-*falciparum* infections in only a small number of cases (12/526), and molecular testing was limited to *P. falciparum*-specific varATS qPCR. As a result, non-*falciparum* or mixed-species infections could not be molecularly confirmed in this study.

**Table 2:** Comparison of the sequential algorithm (decisional and e-algorithm arm) and *Pf*HRP2 results according to the *Plasmodium falciparum* density.

Microscopy (number of parasite/µL)	Standard RDT		Sequential interpretation			Previous antimalarial use in the past 4 weeks (undetermined result)	
	*Pf*HRP2 pos	*Pf*HRP2 neg	Positive	Negative	Undetermined	Ttt+	Ttt-
1–100	0	0	1	0	11	8	3
101–1,000	18	1	13	2	15	12	3
1,001–10,000	26	4	41	2	4	3	1
10,001–100,000	80	1	138	4	2	2	0
>100,000	42	0	105	0	3	2	1
Microscopy positive	**166**	**6**	**298**	**8**	**35**	**27**	**8**
Microscopy negative	**46**	**174**	**6**	**372**	**65**	**57**	**8**
TOTAL number	**212**	**180**	**304**	**380**	**100**	**84**	**16**
Pure gametocytemia	12	1	14	1	10	--	--
*P. falciparum*	165	6	292	11	40	--	--
Other species	2	3	3	3	1	--	--

**Ttt+ =** antimalarial treatment in the last 4 weeks; **Ttt- =** no antimalarial treatment

Among the participants of both intervention arms with a positive sequential diagnosis (*Pf*HRP2 + /*p*LDH+), 98.0% (298/304) were positive by expert microscopy, compared to 78.3% (166/212) who were found positive by routine RDT (*Pf*HRP2+) testing in the control arm. In the intervention arms, 16 cases out of the 100 who had an undetermined sequential diagnosis results did not report previous antimalarial treatment within the past 4 weeks. Half of those (8 cases) were found positive by expert microscopy.

Among participants of both intervention arms with a negative sequential diagnosis (*Pf*HRP2-/*p*LDH-), 97.8% (372/380) were negative by expert microscopy, compared to 96.7% (174/180) found negative with routine RDT (*Pf*HRP2-) of the control arm. Of the 84.0% (84/100) of undetermined sequential diagnosis of both intervention arms who reported an antimalarial treatment within the past 4 weeks, 67.9% (57/84) were negative by expert microscopy. Moreover, 74.3% (26/35) of participant with undetermined diagnostic results had a parasite density below 1,001/µl.

### Accuracy of sequential algorithm and routine *Pf*HRP2 compared to expert microscopy

Table 3 summarizes the diagnostic accuracy of the sequential algorithm of *Pf*HRP2/*p*LDH combined with information on previous antimalarial treatments and routine *Pf*HRP2 tests, compared to expert microscopy as gold standard.

**Table 3:** Accuracy of *Pf*HRP2-RDT and sequential algorithm compared to microscopy (gold standard).

		True positive n (%)	True negative n (%)	False positive n (%)	False negative n (%)	Total
**Sequential diagnostic**	e-algorithm arm	150 (38.4)	223 (57.2)	03 (0.8)	14 (3.6)	**390**
	Decisional arm	156 (39.6)	206 (52.3)	11 (2.8)	21 (5.3)	**394**
	Combined performance	306 (39.1)	429 (54.7)	14 (1.8)	35 (4.4)	**784**
***Pf*HRP2**		166 (42.3)	174 (44.3)	46 (11.8)	06 (1.6)	**392**

Among the participants with conclusive-only results with sequential algorithm (*Pf*HRP2 + /*p*LDH+ and *Pf*HRP2-/*p*LDH-), false positive and false negative rates were 1.2% (8/684) and 0.9% (6/684), respectively. When including cases with initially undetermined diagnostic results that required information on previous antimalarial treatment within the past 4 weeks to confirm malaria status, the overall false positive and false negative rates increased to 1.8% (14/784) and 4.4% (35/784), respectively. In comparison, the routine *Pf*HRP2 RDT in the control arm yielded 11.8% (46/392) false-positive and 1.6% (6/392) false-negative results.

### Diagnostic performance of the sequential algorithm and *Pf*HRP2 with microscopy (gold standard)

The diagnostic accuracy of the sequential algorithm and routine *Pf*HRP2 results compared to expert microscopy (gold standard) are presented in Table 4.

**Table 4:** Diagnostic performance of sequential algorithm and *Pf*HRP2-RDT with microscopy (gold standard).

Accuracy parameters	Routine *Pf*HRP2	e-Algorithm	Decisional algorithm	Both e-Algorithm + decisional algorithm
	% (95%CI)	% (95%CI)	% (95%CI)	% (95%CI)
Sensitivity	96.5 (92.6 - 98.7)	91.5 (86.1 - 95.3)	88.1 (82.4-92.5)	89.7 (86.0 - 92.7)
Specificity	79.1 (73.1 - 84.3)	98.7 (96.2 - 99.7)	94.9 (91.1-97.4)	96.8 (94.7 - 98.3)
Positive predictive value	78.3 (72.1 - 83.7)	98.0 (94.4 - 99.6)	93.4 (88.5 - 96.7)	95.6 (92.8 - 97.6
Negative predictive value	96.7 (92.9 - 98.8)	94.1 (90.3- 96.7)	90.7 (86.2-94.2)	92.4 (89.6 - 94.7)

When the analysis of the sequential diagnostic algorithm was restricted to conclusive results only, sensitivity, specificity, positive predictive value (PPV) and negative predictive values (NPV) were 97.4%, 98.4%, 98.0%, and 97.9%, respectively. However, when undetermined cases were resolved using information on prior antimalarial treatment and included in the analysis, the overall sensitivity decreased to 89.7% while specificity was 96.8%. In comparison, the routine *Pf*HRP2 RDT showed lower specificity (79.1%) and PPV (78.3%), despite comparable sensitivity (96.5%).

To evaluate the impact of seasonality (rainy and dry season) on diagnostic performance, PPV and NPV were stratified by high and low transmission periods (Table 5). As expected, both PPV and NPV varied across seasons, reflecting the prevalence-dependent nature of these metrics. For example, PPV (99.5%) was higher during the peak transmission season (rainy season), while NPV (95.7%) remained high during low transmission season (dry season). These findings indicate that performance estimates for the diagnostic algorithm are context-dependent and may shift with local transmission intensity and seasonal epidemiology.

**Table 5:** Diagnostic accuracy of *Pf*HRP2 versus sequential algorithm by season.

	*Pf*HRP2				Sequential algorithm			
	Sensitivity % (95%CI)	Specificity % (95%CI)	PPV % (95%CI)	NPV % (95%CI)	Sensitivity % (95%CI)	Specificity % (95%CI)	PPV % (95%CI)	NPV % (95%CI)
All	96.5 (92.6 98.7)	79.1 (73.1 - 84.3)	78.3 (72.1 −83.7)	96.7 (92.9 - 98.8)	89.7 (86.0 - 92.7)	96.8 (94.7 - 98.3)	95.6 (92.8 - 97.6)	92.4 (89.6 - 94.7)
**Season**								
Dry	94.4 (86.4 - 98.5)	82.1 (5.1- 87.9)	71.6 (61.4- 80.4)	96.9 (92.2- 99.1)	90.6 (84.4- 94.9)	95.7 (92.8 - 97.7)	90.6 (84.4 - 94.9)	95.7 (92.8 - 97.7)
Raining	98.0 (93.0 - 99.8)	72.5 (60.4 - 82.5)	83.8 (75.8 - 89.9)	96.2 (86.8 - 99.5)	89.2 (84.1 - 93.1)	99.3 (96.1-100.0)	99.5 (97.0 - 100.0)	86.3 (79.9 - 91.2)

*PP*V*: positive* predictive *value; NPV: n*e*gative* predictive *value; Dry season:* November to May*; Rainy season:* June to October.

The agreement between sequential algorithm and expert microscopy was considered to be “very good”; kappa value = 0.871 (95% CI = 0.836–0.906; SE of kappa = 0.035). The agreement between routine *Pf*HRP2 and expert microscopy as gold standard was considered “Good”; Kappa value = 0.737 (95% CI = 0.687–0.786; SE of kappa = 0.049).

### Analysis on discrepancy and undetermined samples

Among false-positive results to routine *Pf*HRP2 RDT, 41.3% (19/46) were confirmed positive by qPCR. In comparison, 50% (7/14) of false positive from the sequential interpretation algorithm combined with information on previous antimalarial treatment within the past 4 weeks were qPCR-positive.

Among participants with undetermined diagnostic results from the sequential diagnostic algorithm, qPCR detected *P. falciparum* DNA in 61.0% (61/100) of cases. Of undetermined cases who reported previous antimalarial treatment within the past 4 weeks, 59.5% (50/84) were qPCR-positive compared to 68.7% (11/16) of qPCR-positive in those without recent treatment.

## Discussion

The study found that several participants who tested positive for malaria with RDT based on the detection of only *Pf*HRP2 were actually not infected with *P. falciparum* when expert microscopy was used as gold standard. The specificity issue of *Pf*HRP2 reported in the study has been documented in several other studies, leading to an overestimation of malaria cases and overlooking the real causes of fever in the event of false positive results for malaria [3,6,9,15–17]. However, the results of the sequential diagnosis of malaria, combining the two-band RDT detecting *Pf*HRP2 and *p*LDH, allowed for differentiating between true and false diagnostic results, which could not be diagnosed with single malaria RDT, and classified them in two distinct groups: conclusive-only results (with a diagnostic accuracy over 97%), and undetermined results for which an additional diagnostic investigation is required. The classification of diagnostic results into these two groups allowed healthcare workers to take an appropriate treatment decision for 87% of patients and avoided unnecessary use of antimalarials and subsequently antibiotics. However, the proportion of undetermined (*Pf*HRP2 + /*p*LDH-) results observed in this study is clinically relevant and reflects known limitations of antigen-based diagnostics in settings of recent treatment, low parasite density, or recrudescence/reinfection. While exclusion of undetermined results yields high diagnostic performance, inclusion of these cases, resolved using treatment history, results in a meaningful reduction in sensitivity, highlighting a trade-off between conclusive classification and the risk of missed active infections. The relative improvements of malaria diagnosis by the use of information of prior antimalarial treatment can be failed by drug resistance [18,19].

The duration of previous antimalarial treatment may appear to be sufficiently extensive for parents or guardians to accurately recall. Since failing to treat sick people could result in ongoing disease transmission, false negative results are quite concerning. The “undetermined” (*Pf*HRP2 + /*p*LDH-) category reflects well-recognised biological and analytical limitations of *p*LDH-based rapid diagnostic tests, which exhibit reduced sensitivity at low parasite densities compared with *Pf*HRP2-based assays [1,20]. In high-transmission settings, this category may also include cases of recent *Plasmodium* reinfection or persistent *Pf*HRP2 antigen following prior antimalarial treatment, highlighting the operational challenges of interpreting these results [18,19]. Consequently, this category includes true infections characterized by low parasitaemia as well as cases with persistent *Pf*HRP2 antigen following recent treatment. Consistent with this interpretation, a substantial proportion of undetermined cases occurred in lower parasite density strata, and many children in this group remained qPCR-positive. qPCR was not considered as the reference test in this study, because it may detect a sub-microscopic infection (reflecting asymptomatic carriage rather than clinical symptoms). Therefore, qPCR-positive/microscopy negative discrepant cases should be interpreted with caution, as qPCR may detect low-density parasitemia or residual parasite DNA following recent infection or treatment [21]. These observations indicate that resolving undetermined results solely based on information on previous treatment, including seasonal malaria chemoprevention (SMC) is inherently limited and may lead to misclassification of low-density, recrudescent, or reinfection cases. Althought SMC is implemented in the study area in accordance with national policy during periods of high transmission (July to October), information on prior antimalarial treatment, including potential participation in SMC, was collected by interviewing thecaregivers. This represents an important limitation of the proposed diagnostic approach and underscores the need for cautious interpretation or the use of more accurate diagnostics that can be feasibly implemented in remote settings. Additionally, the *Pf*HRP2-/*p*LDH+ pattern is commonly used to suggest a possible non-*falciparum* malaria or *P. falciparum* infection with potential *hrp*2 gene deletion. However, the present study was not designed to robustly evaluate these scenarios. Non-*falciparum* infections were rarely detected by microscopy, and molecular testing relied on a *P. falciparum*-specific assay, precluding confirmation of non-*falciparum* or mixed-species infections. In line with WHO guidance, such results should therefore be considered indicative rather than confirmatory, particularly in settings where species distribution is uncertain or mixed infections may occur.. This study also reported a discrepancy between microscopy and qPCR results among patients with undetermined results who reported recent antimalarial treatment. This finding may be explained by the higher analytical sensitivity of qPCR, which can detect low parasite density or residual parasite DNA following a (successful) antimalarial treatment, whereas microscopy has a higher detection threshold and may yield negative results in such cases [22,23].

The performance of a diagnostic test depends on the number of incorrect results [24]. In this study, the routine RDT showed a higher sensitivity (>95%) for the detection of the *P. falciparum* parasite than the sequential diagnostic algorithm (<90%). However, the sequential diagnosis showed a higher specificity (>95%) in the detection of *P. falciparum* parasites. This may be due to the inaccurate data on previous antimalarial treatments reported by caregivers [5]. Indeed, the previous antimalarial treatments collected in this study were those prescribed by a health professional at healthcare centers, and those received during the seasonal malaria chemoprevention (SMC) campaign during the period of high transmission. Unfortunately, the clinical team did not have any evidence that the previous treatments were supplied and provided correctly. While the results of the routinely used *Pf*HRP2 RDT are similar to those reported by Kiemde et *al*. in 2019, the results of the sequential diagnosis are different [5]. These differences observed for the sequential diagnosis could be explained by the SMC campaign during the study period, and the duration of previous treatment collected (4 weeks in the present study *versus* 2 weeks in the 2019 study). Given the proportion of participants requiring confirmation of malaria infection (approximately 13%), the sequential diagnosis could be used to quickly triage real malaria cases. However, failing to treat sick people among undetermined cases could result in ongoing disease transmission due to false negative results in this group. In this study, confirmation of undetermined cases based solely on prior antimalarial treatment demonstrated clear limitations.

It would be necessary to confirm the diagnosis of malaria in undetermined cases with a more efficient test that could be deployed in rural areas. In this study, qPCR was not used as a reference test, given the fact that submicroscopic infection can be detected, which may not correlate with observed clinical symptoms [6]. In contrast to our initial hypothesis, which stated that using information on previous treatment would have allowed us to better distinguish low-grade parasitaemia from the persistence of the *Pf*HRP2 antigen, we discovered that approximately 60% of children who reported previous antimalarial treatment had a positive qPCR. These findings differ from those reported in our previous study and by Maltha et *al*. [5,6] This could be explained by poor medication delivery during health center consultations and chemoprevention bulk antimalarial distribution (administration not monitored) or a wrong report on previous antimalarial treatment, as no resistance to the antimalarials administered was detected in our research area [25]. Furthermore, our findings indicate that the sequential algorithm included undetermined results corrected yielded a higher false-negative rate than the *Pf*HRP2-based RDT. This represents a limitation of the proposed algorithm, as undetermined cases are classified based on information about prior antimalarial treatment without dispensing, adherence, and timing confirmed, misclassification cannot be excluded. The delay of treatment in false negative cases may lead to disease progression and contribute to the continued transmission of malaria.

PPV and NPV are inherently dependent on the prevalence of infection and therefore vary with seasonal transmission intensity and local epidemiology. Our stratified analysis demonstrates that PPV is higher during peak transmission periods and lower during low transmission periods, and vice versa for NPV. Accordingly, claims of improved predictive values should be interpreted within the specific epidemiological context of this study rather than as universally generalisable. These findings highlight the importance of considering seasonality and transmission dynamics when applying or interpreting algorithm-based malaria diagnostics in different settings.

## Conclusion

This study confirms the usefulness of the sequential algorithm for enhancing specificity and facilitating cases stratification of clinical malaria in endemic areas. However, using the history of antimalarial treatment within the past four weeks as a decision-support tool for the classification of undetermined cases in endemic settings introduces a limitation as it may decrease the sensitivity of the algorithm. A more efficient (diagnostic) tool, suitable for deployment in rural areas, would be necessary to confirm malaria in these situations.
